# Fusarium Head Blight Control and Prevention of Mycotoxin Contamination in Wheat with Botanicals and Tannic Acid

**DOI:** 10.3390/toxins6030830

**Published:** 2014-02-26

**Authors:** Hans-Rudolf Forrer, Tomke Musa, Fabienne Schwab, Eveline Jenny, Thomas D. Bucheli, Felix E. Wettstein, Susanne Vogelgsang

**Affiliations:** Agroscope, Institute for Sustainability Sciences, Reckenholzstrasse 191, 8046 Zurich, Switzerland; E-Mails: tomke.musa@agroscope.admin.ch (T.M.); eveline.jenny@agroscope.admin.ch (E.J.); thomas.bucheli@agroscope.admin.ch (T.D.B.); felix.wettstein@agroscope.admin.ch (F.E.W.)

**Keywords:** *Fusarium graminearum* (FG), antifungal, natural compound, phenolic, phytoalexin, elicitor, deoxynivalenol (DON), forecasting, organic

## Abstract

Suspensions or solutions with 1% of Chinese galls (*Galla chinensis*, GC) or 1% of tannic acid (TA), inhibited germination of conidia or mycelium growth of *Fusarium graminearum* (FG) by 98%–100% or by 75%–80%, respectively, whereas dried bark from buckthorn (*Frangula alnus*, FA) showed no effect at this concentration. In climate chamber experiments where the wheat variety “Apogee” was artificially inoculated with FG and F. crookwellense (FCr) and treated with 5% suspensions of TA, GC and FA, the deoxynivalenol (DON) content in grains was reduced by 81%, 67% and 33%, respectively. In field experiments with two commercial wheat varieties and artificial or semi-natural inoculations, mean DON reductions of 66% (TA) and 58% (FA), respectively, were obtained. Antifungal toxicity can explain the high efficacies of TA and GC but not those of FA. The Fusarium head blight (FHB) and mycotoxin reducing effect of FA is probably due to elicitation of resistance in wheat plants. With semi-natural inoculation, a single FA application in the first half of the flowering period performed best. However, we assume that applications of FA at the end of ear emergence and a treatment, triggered by an infection period, with TA or GC during flowering, might perform better than synthetic fungicides.

## 1. Introduction

Contamination of food and feed with mycotoxins is a major concern for growers and industry in small grain cereals and especially in maize production. Globally, *Fusarium graminearum* (FG) Schwabe (teleomorph *Gibberella zeae*) is the most prevalent Fusarium head blight (FHB) causing fungus and the main source of deoxynivalenol (DON) and zearalenone (ZEA) contamination of wheat [1], most probably because of its high genetic diversity and an increasing surface of maize cropping. The key factors of *F. graminearum* (FG) infections in wheat are maize or wheat as a previous crop, reduced or zero tillage and susceptible wheat varieties [2,3]. The reasons for increasing cases of such high risk situations are mostly of economical nature. In Switzerland, even by using the lowest susceptible wheat variety and intensive mechanical maize residue mulching treatments, the DON contamination has not often been reduced below the maximum limit of 1.25 mg kg^−1^ in unprocessed cereals [4] when wheat following grain maize was sown with reduced or zero tillage [5]. For control of FHB, the application of a fungicide during wheat anthesis (growth stage [GS] 61–69; Zadoks [6]) is often recommended. In field trials in the UK by Edwards and Godley [7], applications of the prothioconazole product Proline^®^ at GS 65 resulted in an FHB and DON reduction in wheat of nearly 60%. In own field trials with artificial infections with *F. culmorum* and the application of triazole fungicides at GS 57 and 67, we observed DON reductions of 29% and 71%, respectively [8]. Apart from the choice of product to optimise the efficacy of a treatment, the timing of the application based on a forecasting system such as FusaProg [9] is helpful for sufficient control of FHB.

In European countries, except UK, FHB and DON contamination in organic wheat production is generally considered as less important as in conventional production [10,11,12]. With the trend to reduced soil conserving tillage and expanding maize cropping in organic wheat production [13,14], increasing problems must be expected and natural fungicides could help to reduce the risk of mycotoxin contaminations [15]. Botanicals and other natural antifungal agents have been shown to inhibit *Phytophthora infestans*, the cause of potato late blight [16,17,18,19], *Microdochium majus*, the cause of snow mold in wheat [20] or to be effective against food contaminating fungi such as *F. oxysporum*, *Alternaria alternata* und *Aspergillus* species [21]. With respect to choice of natural antifungal compounds, botanicals used in Chinese medicine and known for antioxidant and antimicrobial activity could be promising. For example, *Rheum palmatum* (RP) L. (Chinese rhubarb), *Frangula alnus* (FA) Mill. (buckthorn bark) and *Galla chinensis* (GC; Chinese gallnuts) are all rich in tannins and other phenolic compounds [19,20,22,23]. Tannins and tannic compounds are used in dietary and medicinal herbs with antioxidant and antimicrobial activity and have also been considered to prevent cancer [24,25,26]. As early as in 1913, Knudson [27] reported that tannic acid (TA; C_76_H_52_O_46_) is even at low concentrations toxic to a large number of fungi. Furthermore, strong inhibition was also observed towards bacteria [28,29]. Antibiotic phenolic compounds were found in many plants and play constitutively or induced by elicitors a crucial role in the defense of plant diseases [30]. Examples of such induction of antibiotic phenolics in wheat are seed treatments with Chitosan^®^, a product based on chitin, and silicon [31,32]. Seed treatments with Chitosan^®^ reduced seed borne FG incidence by more than 50% whereas silicon sprayed on wheat plants induced the formation of phenolics which in turn reduced powdery mildew (*Erysiphe graminis*) incidence on wheat leaves.

The main objective of this study was to evaluate the potential of TA and the botanicals GC, RP and FA to reduce head blight caused by FG and the DON concentration in integrated and organic wheat production. The particular aims were to investigate the effect of the antifungal botanicals (ABs): (1) on *in vitro* conidia germination and mycelial growth; (2) on FHB in wheat through FG and *F. crookwellense* (FCr) as well as DON and nivalenol (NIV) contamination in climate chamber; and (3) in field experiments from 2006 to 2010.

## 2. Results and Discussion

### 2.1. Isolate Specific Inhibition of Conidia Germination with TA

**Figure 1:**
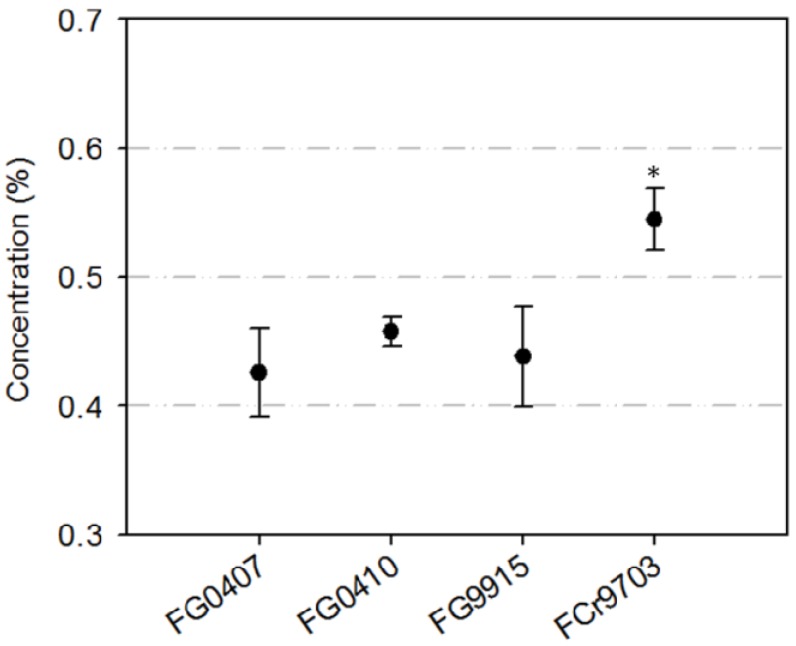
*In vitro* experiment: isolate specific inhibition of conidial germination with tannic acid (TA): concentration of TA needed for 50% inhibition (EC_50_) of conidia germination from three *Fusarium graminearum* (FG) and one *F. crookwellense* (FCr) isolate/s. Mean values of the germination of each 60 conidia and the standard error of means. A “*” indicates a significant difference from the other isolates, according to a one way analysis of variance (ANOVA) and a Holm-Sidak post hoc test at *p* < 0.05.

Based on re-isolations of *Fusarium* species from wheat grains of our first field experiment, we realised that we did not investigate four isolates of FG as originally planned, but with three FG and one FCr isolate. Since the FCr isolate is of Swiss origin and a potent NIV producer, we decided to continue with this combination of strains and both *Fusarium* species. The mean rates of conidia germination of the three FG isolates FG0407, FG0410, FG9915 and the FCr isolate FCr9703 (all single conidia) in the control treatment with water were 86%, 79%, 82% and 97%, respectively. With 0.19% Pronto^®^ Plus (PrP), zero germination was observed from the isolates FG0407 and FG0410 and low germination rates of 7% and 1% for FG9915 and FCr9703, respectively. Corrected for a germination rate of 100% of the isolates in the control treatments, the effective concentration for 50% inhibition (EC_50_) through TA at 0.125%, 0.25%, 0.5% and 1% was calculated. The EC_50_ with TA and the three FG isolates varied little between 0.43% and 0.46%. The EC_50_ for the FCr isolate was 0.55%, making it slightly, but nonetheless, significantly (*p* < 0.05) higher (Figure 1).

The EC_50_ with TA for the FG and FCr isolates was about 10 times higher than those reported for *Colletotrichum lindemuthianum* with extracts of cascalote (*Ceasalpinia cacalaco*) [33]. Cascalote is also a source for phenolics such as gallic and tannic acid. However, for *Microdochium majus* and suspensions with 0.1%–1.0% GC, similar efficacies as with TA and FG were observed [20].

### 2.2. Inhibition of Conidia Germination of FG0407 with TA and Botanicals

**Figure 2:**
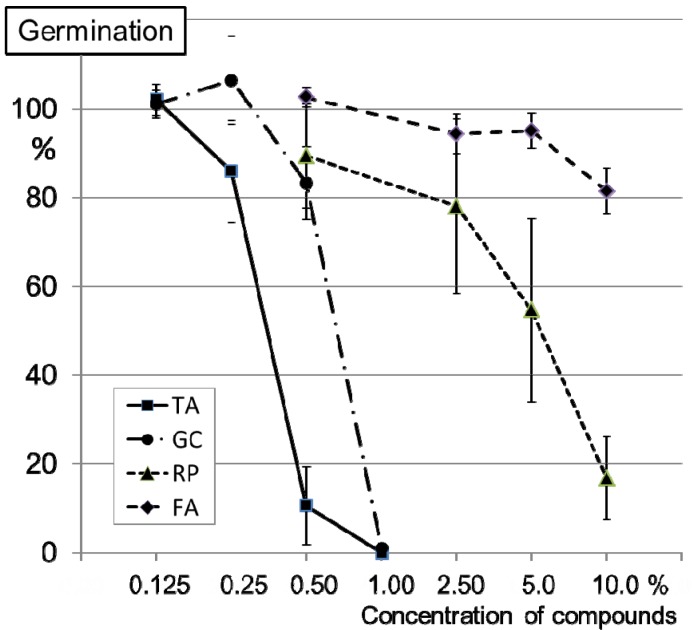
*In vitro* experiment: conidia germination of FG0407 with various concentrations of TA and botanicals. Solutions or aqueous extracts of TA and the botanicals *Galla chinensis* (GC), *Rheum palmatum* (RP) and *Frangula alnus* (FA) were applied. The control treatment with water was set to 100% germination. Each data point represents the relative mean of the germination of 90 conidia. Bars indicate the standard error of mean.

Based on the results and the narrow EC_50_ band for all isolates, we subsequently restricted the comparison of the conidia inhibiting effect of the three ABs and TA to one isolate, FG0407. Although it is not known whether all four isolates would react similar to FG0407, this isolate proved to be pathogenic and toxigenic in preliminary climate chamber trials. An application of PrP at 0.19% completely inhibited the germination of FG0407 (data not shown). Complete inhibition was also observed with TA and GC at 1%. Almost no effect was observed with the RP and the FA extracts at 1%. Elevated concentrations with 10% RP or FA reduced the germination rate down to about 20% or 80%, respectively (Figure 2).

### 2.3. Inhibition of Mycelial Growth of FG0407 with TA and Botanicals

**Figure 3:**
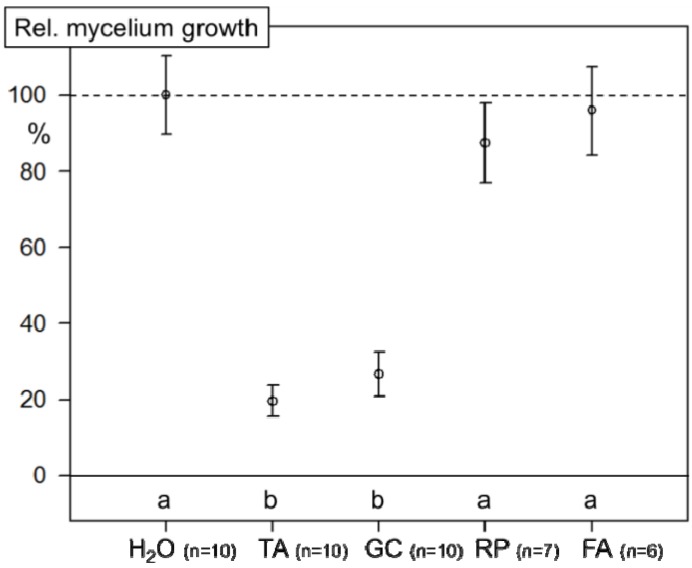
*In vitro* experiment: mycelial growth of FG0407 with TA and botanicals. Solutions of TA and suspensions of 1% of GC, RP and FA were applied on agar (2 mL suspension per Petri dish). The control treatment with water was set to 100% radial growth. For treatments labeled with the same letter, mean values are not statistically different (*p* < 0.05) according to a Tukey test.

Application of 2 mL of 1% TA and 1% GC suspensions in agar in Petri dishes significantly (*p* < 0.001) inhibited the mycelium growth of FG0407 by 80% or by 73%, respectively. A slight but not significant inhibition (13%) resulted with the RP treatment and almost none was observed after a treatment with FA (Figure 3).

In preliminary trials, we observed that agar solidification was not successful when concentrations of TA greater than 0.5% were incorporated. Therefore, all antifungal agents, including TA solutions, botanicals and PrP were applied onto the surface of the already solidified agar. The aqueous solutions were absorbed by the agar or were forced to evaporate in an air stream at room temperature. Certainly, with this procedure adapted for TA and our ABs, a direct comparison with other investigations employing agar incorporated agents is not possible. With our approach we can only estimate the EC of the agents needed to sufficiently reduce mycelial growth. Still, the primary aim of this experiment was to compare the effect of the selected agents and not to determine absolute values for toxic concentrations. If we assume that the 2 mL 1% GC poured on 20 g agar in our experiment (~45% inhibition of FG) is distributed in the agar, the concentration corresponds approximately to those of 0.1% GC incorporated in agar for an assay with *M. majus* (~70%) [20]. Hence, we assume that the experimental set-ups are comparable. Nevertheless, a direct comparison is doubtful since different fungal species were investigated. This might explain the contrasting results with RP in the current study and those from an investigation with *P. infestan*s: in our experiment with 1% RP, hardly no effect was observed, whereas in the study by Hu *et al.* [17], concentrations of 0.4% RP incorporated into the agar completely inhibited mycelial growth of *P. infestan*s [17].

### 2.4. Climate Chamber Experiment—Reduction of Disease Severity and Mycotoxins in Artificially Inoculated Wheat

In these experiments, the effect of antifungal agents was examined on the artificially inoculated wheat cultivar “Apogee”. A mixture of the same isolates as *in vitro* was used for the inoculation. Since the preparations with TA and the botanicals were acid, a tap water and acidified tap water control were used (Table 1).

**Table 1:** Description of treatments in climate chamber experiments with the spring wheat cultivar “Apogee” and in field experiments with the winter wheat cultivars “Runal” and “Levis”. Type of experiments: A: climate chamber experiments; and B: field experiments with artificial inoculations (2006 and 2008–2010).

Treatment Nb.	Ingredient	Identifier	Application *vs.* inoculation		Concentration	pH	Type of experiment	
			before	after	%	±0.2	A	B
1	Tap water (control 1)	Water	×	-	-	7.8	×	×
2	Acidified water (c. 2)	ac-Water	×	-	-	4.0	×	-
3	Tannic acid	TA b i.	×	-	5	3.8	×	×
4	Tannic acid	TA a i.	-	×	5	3.8	×	-
5	Tannic acid	TA b+a i.	×	×	5	3.8	×	×
6	*Galla chinensis*	GC b+a i.	×	×	5	3.9	×	×
7	*Rheum palmatum*	RP b+a i.	×	×	5	5.0	×	×
8	*Frangula alnus*	FA b+a i.	×	×	5	5.2	×	×
9	Pronto^®^ Plus	PrP b i.	×	-	0.375	8.2	×	×

b i./a i.: before/after inoculation; b+a i.: application one day before and after inoculation; pH: pH of water, suspensions with botanicals and PrP.

**Figure 4:**
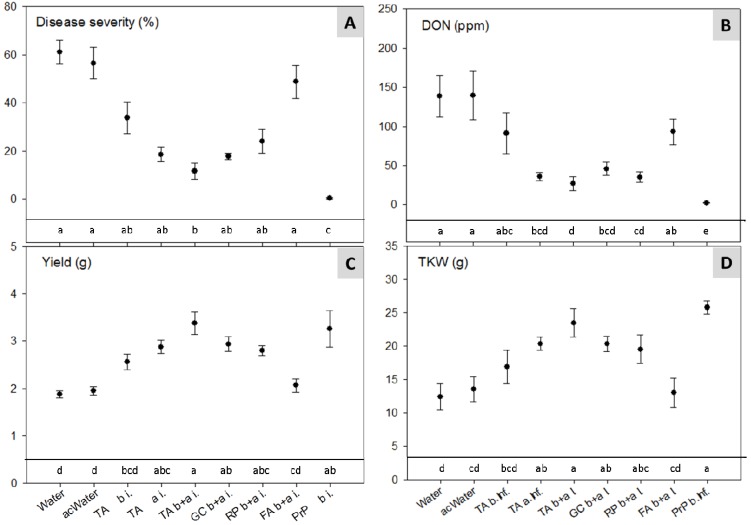
Climate chamber experiment: Effect of treatments with TA, GC, RP, FA and Pronto Plus^®^ (PrP) on: (**A**) disease severity (% area with FHB symptoms); (**B**) deoxynivalenol (DON) content; (**C**) yield of three heads; and (**D**) thousand kernel weight (TKW) of the spring wheat cultivar “Apogee” after artificial inoculation with a mixture of three *F. graminearum* (FG) and one FCr isolate/s. Data are pooled results from two experiments with four replicates of each treatment. For treatments labeled with the same letter, mean values are statistically not different according to a Tukey test (*p* < 0.05). Error bars indicate the standard error of mean. Treatment abbreviations are as in Table 1.

The artificial inoculation had a strong effect on all evaluated parameters. The best effect on disease inhibition and DON reduction was achieved with PrP resulting in a complete elimination of symptoms and 98% less DON compared with the control treatment (Figure 4). A significant effect (*p* < 0.05) was also observed with TA applications before and after inoculation (TA b+a i.) with a reduction of the disease severity from 59% down to 12% (Figure 4A). All botanicals, with the exception of FA and the treatment with TA before infection, reduced the DON contamination in the grains significantly by 67% to 80% (Figure 4B). An even stronger effect was observed for the yield and the thousand kernel weight (TKW). The “TA b+a i.” treatment performed as excellent as PrP and gave a 77%–80% higher yield compared with the control treatments (Figure 4C,D). There was a strong correlation between disease, yield, TKW and DON (Table 2). In fact, the Spearman correlation coefficients between disease and DON were as high as 0.948 (*r*^2^ = 0.90) (Table 2). Such strong relationships were also reported for the incidence of *F. poae* in grains and the NIV content in field experiments with artificial inoculations of different cultivars [34]. In the current study, the correlation between disease severity and NIV was substantially lower but still very high (*r*^2^ = 0.77). The NIV and AcDON contents were analysed only in one of the two climate chamber experiments. In this experiment, the *r*^2^ for DON and AcDON was 0.89, a strong indication that AcDON was produced by the FG isolates. Between NIV and DON, the *r*^2^ was still remarkable with 0.57. Therefore, it can be assumed that NIV was produced by an important fraction of FG isolates and not only by the FCr isolate as originally expected. In these climate chamber experiments, the amount of harvested seed was only sufficient to analyse the mycotoxins but not to perform a seed health test.

In contrast to other studies showing good efficacies against potato late blight under field conditions [19], no significant effect was observed with buckthorn bark (FA) in the “Apogee” experiments. However, this result was not surprising since there was no effect of FA on *in vitro* FG conidia germination or on mycelial growth (Figure 2 and Figure 3). Nevertheless, the good efficacy obtained with Chinese rhubarb (RP b+a i.), which showed little effects *in vitro*, but an *in vivo* performance that was comparable with the gallnut suspension (GC b+a i.) with strong effects *in vitro*, was not expected. One possible reason for this phenomenon could be an elicitation of defense mechanisms. For example, in experiments with RP and FA against downy mildew (*Plasmopara viticola*) on grapes (*Vitis vinifera*), a strong induction of resveratrol and other stilbenoids (polyphenols) was found [35]. In these experiments, TA and GC completely inhibited infections by *P. viticola*, but they did not induce the production of stilbenoids.

**Table 2:** Climate chamber experiment: Correlation between disease severities (% area with FHB symptoms), yield and mycotoxin contamination with data from two experiments with the spring wheat cultivar “Apogee”. Analysis for nivalenol (NIV) and acetylated deoxynivalenol (Ac-DON) were conducted only in the second experiment. The Spearman correlation coefficients were all with *p* < 0.001; coefficients with *r* > 0.8 are indicated in bold.

Parameter	TKW (*n* = 72)	Disease severity (*n* = 72)	DON (*n* = 72)	NIV (*n* = 36)	AcDON (*n* = 36)
Yield	0.660	−0.737	−0.637	−0.560	−0.675
TKW	-	−**0.919**	−**0.932**	−0.664	−**0.814**
Disease severity	-	-	**0.948**	**0.877**	**0.907**
DON	-	-	-	0.752	**0.941**
NIV	-	-	-	-	0.786

### 2.5. Field Experiments with Artificial Inoculation—Reduction of FHB and Mycotoxins in Wheat

In 2006 and from 2008 to 2010, field experiments with artificial inoculations were performed with the two winter bread wheat cultivars “Runal” and “Levis”. Artificial inoculations were in general conducted near mid anthesis (GS 63–65) together with applications for FHB control one day before and/or after the fungal inoculation. For logistic reasons, however, both cultivars were inoculated at the same day.

**Figure 5:**
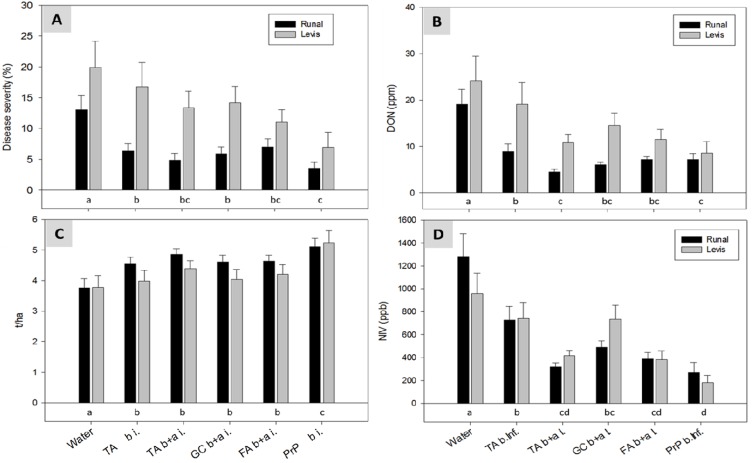
Field experiments with artificial inoculation: Effect of treatments with TA, GC, FA and PrP: (**A**) on the area of heads with FHB symptoms (disease severity); (**B**) on DON content; (**C**) on yield; and (**D**) on NIV content of the winter wheat cultivars “Runal” and “Levis” after artificial inoculation with a mixture of three FG and one FCr isolate/s. Data are pooled results from field experiments located at Zurich-Reckenholz in 2006 and from 2008 to 2010 (for choice of years, see Experimental Section). Bars with means of 16 values (four years and four replicates) and standard error of means. For treatments labeled by the same letter, mean values are statistically not different according to Tukey test (*p* < 0.05).

All treatments with antifungal compounds caused significant effects on FHB disease symptoms on wheat heads, yield and mycotoxin accumulation. The fungicide PrP showed the best performance and resulted in the highest yield (*p* < 0.05) with a mean increase of 37% for both cultivars compared with the water control throughout the four experimental years (Figure 5C). With the botanicals and TA, yield increases of 13%–23% (TA a+b i.) were achieved. With respect to FHB on heads (disease severity; Figure 5A), DON and NIV (Figure 5B,D), the TA treatment “TA b+a i.” was as effective as PrP.

The higher yield caused by the synthetic fungicide could be explained by its broad spectrum for disease control. Apart from the excellent performance of TA and GC under field conditions, the efficacy of buckthorn bark (FA) was also highly remarkable, since preparations of FA were not only ineffective in the *in vitro* experiments but also in the climate chamber experiments with the cultivar “Apogee” (Figure 4). This finding may be explained by induction of self defense mechanisms with production of phenolic compounds as observed in the mentioned experiment on downy mildew of grapes [35] or with Chitosan^®^ which reduced seed-borne FG by inducing the formation of phenolic acids and lignin [29]. The function of induced or constitutive phenolic compounds in disease resistance has been described for diverse pathogen-host interactions [30,36] and phenolic compounds may also play a role as resistance factors in the FHB wheat interaction [37]. The assumed lack in induction of FHB resistance in the cultivar “Apogee” through FA could be based on its overall high susceptibility to various diseases and a deleted QTL region of the chromosome 3BS FHB [38].

High correlations were observed for FHB severity on heads *versus* yield, FHB severity *versus* DON and *versus* NIV content as well as for yield *versus* DON and *versus* NIV content (Table 3). The high correlation between DON and NIV (*r* = 0.893) together with that between the FG incidence in grains and NIV indicates that NIV was also produced by the poly conidia FG isolates and not only by FCr.

**Table 3:** Field experiments with artificial inoculations in 2006, 2007, 2009 and 2010 (for choice of years, see Experimental Section 3.6): Correlation between yield, FHB (% area with symptoms on heads) and mycotoxin concentrations. Spearman correlation coefficients for 178 observations and symbols for significance, NS: not significant, * = *p* < 0.05, ** = *p* < 0.01, and *** = *p* < 0.001. Coefficients with *r* > 0.8 are indicated in bold. ZEA: zearalenone.

Parameter	Disease severity	FG incidence	FCr incidence	DON	ZEA	NIV
Yield	−**0.868**	−0.680	−0.168	−**0.814**	−0.112	−**0.836**
	***	***	*	***	NS	***
Disease severity (FHB)	-	0.703	0.117	**0.879**	0.333	**0.849**
		***	NS	***	***	***
FG incidence	-	-	0.228	0.775	0.348	0.637
	-	-	**	***	***	***
FCr incidence	-	-	-	0.254	0.133	0.370
	-	-	-	**	NS	***
DON	-	-	-	-	0.475	**0.893**
	-	-	-	-	***	***
ZEA	-	-	-	-	-	0.316
	-	-	-	-	-	***

### 2.6. Field Experiments with Semi-Natural Inoculation—Reduction of FHB and Mycotoxins in Wheat

**Figure 6:**
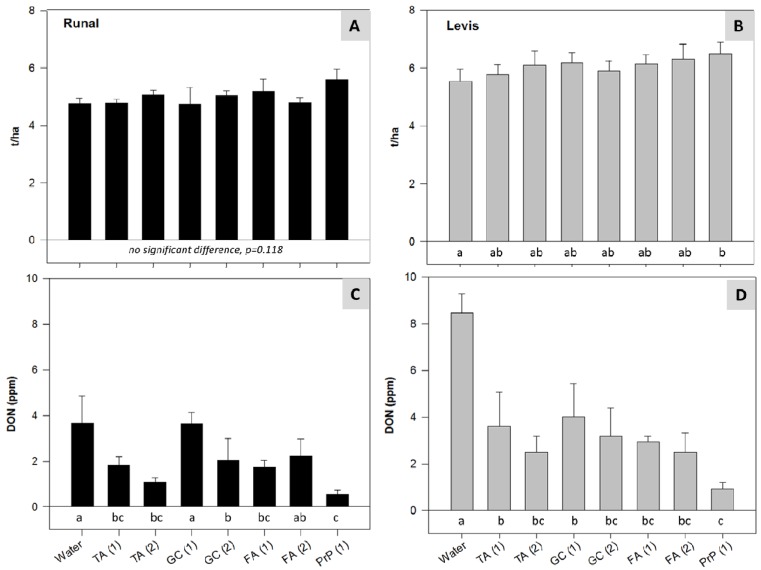
Field experiment with semi-natural inoculation (2010): Effect of treatments with TA, GC, FA and PrP on (**A**,**B**) yield and (**C**,**D**) DON content, in the winter wheat cultivars “Runal” and “Levis”. (1): application on 7 June 2010; (2): applications on 7 and 8 June 2010. Data from field experiment at Zurich Reckenholz with infections originating from FG/FCr infected maize stubbles. Bars with mean values and standard error of means. For treatments labeled by the same letter, mean values are statistically not different according to Tukey test (*p* < 0.05).

In commercial wheat fields with maize as the pre crop and zero or reduced tillage situations, DON values exceeded often the EU limit of 1.25 ppm in unprocessed cereals, even when growing varieties with low FHB susceptibility [5]. Field experiments with semi-natural inoculation represent an approach to mimic conditions as in commercial wheat fields. Such a field experiment was conducted in 2010. Our DON forecasting system FusaProg [9] was employed in order to optimise the timing of the applications. In the first half of wheat flowering, the system forecasted FG infection periods for 6 and 7 June 2010. Therefore, the treatments were applied as soon as possible on 7 and 8 June 2010. In the second half of the flowering period, no additional infection periods were registered. The fungicide PrP significantly increased the yield of “Levis” but not that of “Runal” (Figure 6A,B). For “Runal”, all treatments except those with application of GC (1) and FA (2) significantly reduced the DON content (Figure 6C). For Levis, all treatments significantly reduced the DON content (Figure 6D). The good efficacy of a single application FA (FA (1); Figure 6C,D) on “Levis” and on “Runal” can again be best explained with the induction of plant defense mechanisms [35]. Differences in the efficacy between the two cultivars might be a result of different defense induction abilities of cultivars and/or effects of the growth stage as it has been reported in other investigations with the crops wheat and grapevine [29,37].

## 3. Experimental Section

### 3.1. Fungal Isolates, Growth Conditions and Antifungal Agents

For *in vitro*, *in vivo* and field experiments, three isolates of FG, FG9915 (CBS 121291; CBS: Centraalbureau voor Schimmelcultures), FG0407 (CBS 121296), FG0410 (CBS 121292) and one isolate of FCr, FCr9703 (CBS 121293), were used. All isolates originate from wheat grains from eastern and midland regions of Switzerland. Starter cultures and fungal inoculum was produced in 9 cm diameter Petri plates containing autoclaved (20 min, 121 °C) potato dextrose agar (PDA; 39 g L^−1^, CM0139; Oxoid Ltd., Hampshire, UK). After inoculation, plates were incubated for 6–7 days at 19 ± 1 °C with a photoperiod of 12 h dark/12 h near-ultraviolet light. Conidia suspensions for *in vitro* tests, artificial infections in climate chambers and in the field were obtained by washing off the conidia from the cultures with deionised water with 0.125‰ Tween^®^20 (Sigma Aldrich, Buchs, Switzerland). Conidia concentrations were measured and adjusted to the desired concentration.

The following ABs were used: dried bark of FA (Frangulae corticis sicc norm), dried root of RP (Rhei radix pulv) both from Hänseler AG (Herisau, Switzerland), powder of GC galls (origin: Sichuan, China; purchased from Berg-Apotheke, Zurich, Switzerland) and the polyphenol TA (tannic acid powder, puriss; Sigma Aldrich). The plant material was finely ground with a centrifugal mill (mesh size 0.08 mm; Retsch ZM 200, Schieritz & Hauenstein AG, Arlesheim, Switzerland). The selection of the ABs was based on promising botanicals from earlier experiments, including investigations with *Phytophthora infestans* and *Microdochium majus* [17,18,19,20]. According to the results, GC was one of the best performing botanicals. In China, GC galls from *Rhus chinensis* Mill., induced by larvae of the aphid *Melaphis chinensis*, serve as raw material for industrial production of TA, hence, this compound was also integrated in the experiments of this study [17]. This high molecular weight polyphenolic has a broad field of applications and, for example, is used in human medicine for inhibition of melanogenesis in melanoma cells [39] and also for antidiarrheal effects [40]. The fungicide Pronto Plus^®^ (PrP; active ingredients 25.5% spiroxamine, 13.6% tebuconazole) was integrated to compare the efficacy of the antifungal agents with good performing synthetic fungicides to control FG in wheat [7].

### 3.2. Isolate Specific Inhibition of Conidia Germination with TA

Microscope slides (76 mm × 26 mm) were placed in Petri plates onto moistened (2 mL sterile deionised water) filter papers (diameter 8.5 cm, Nr. 591, Schleicher & Schuell, München, Germany) and three water agar plugs (1 cm diameter) were placed on each slide. Each treatment consisted of two Petri plates, resulting in a total of six agar plugs. One droplet of 15 µL TA with concentrations of 0.125%, 0.25%, 0.5% and 1% were pipetted onto each agar plug and were allowed to evaporate for 20 min in a sterile bench. Sterile, deionised water served as the control treatment. Subsequently, for each isolate, one droplet consisting of 15 µL of the conidial suspensions with 3.3 × 10^4^ conidia mL^−1^ was applied to the plugs. Petri plate lids were closed and plugs were incubated for 24 h at 10 °C and 70% relative humidity (RH) in the dark. Conidia were killed and stained with one drop of a solution with 0.19% PrP and 0.5% cotton blue. The germination rate was assessed on each plug with the aid of a light microscope (400 magnification) by determining the ratio of germinated conidia from a total of 30 conidia within three different visual fields. A conidium was assigned as germinated when the germination tube was longer than the width of the conidium. Based on the results from the five concentrations with six agar plugs, the concentration for a 50% inhibition of the germination of the conidia (EC_50_) was calculated with the software ED50plus v1.0 [41]. The experiment was conducted twice and the results from the two experimental runs were pooled.

### 3.3. Inhibition of Conidia Germination of FG0407 with TA and Botanicals

Using a single conidium isolate of FG0407, we examined the efficacy of the ABs and the fungicide PrP. Preliminary trials showed that germinating conidia are hardly visible on agar containing powder particles. Hence, for this experiment, aqueous extracts of the botanicals were used as opposed to suspensions from botanicals in the *in vivo* and the field experiments. For each of the three botanicals, 10 g powder were suspended in 100 mL autoclaved deionised water and stirred for 3 h at ambient temperature. The aqueous extracts were subsequently filtered using fluted filters (diameter 15 cm, 520 A 1/2, Schleicher & Schuell).

TA and GC were tested at 0.125%, 0.25%, 0.5% and 1% (w/v). RP and FA were tested with aqueous extracts of 0.5%, 2.5%, 5% and 10% (w/v). The elevated concentrations for RP and FA were used, since in preliminary trials almost no effect was observed with RP and FA at 1%. Sterile, deionised water served as the control treatment. For each treatment, the conidia germination rate was determined as described above. The germination rate of the control treatment was set to 100% and the results from the other treatments were adjusted correspondingly. The experiment was repeated three times and the results from the three experimental runs were pooled.

### 3.4. Inhibition of Mycelial Growth of FG0407 with TA and Botanicals

Schott flasks with autoclaved PDA medium were placed in a water bath (60 °C) and while stirring amended with streptomycin sulphate (0.1 g L^−1^). For each Petri plate, 20 g of agar was poured into Petri plates. After one to two days, 2 mL water (control), water with 1% powder of ABs or with TA were evenly spread over the agar surface with a sterilized spreader rod. Subsequently, the Petri dishes were opened and placed into a sterile bench for 2–3 h until the weight of the agar was reduced back to 20 g. For each treatment, five Petri plates were used. Using a cork borer, mycelial plugs (diameter 0.5 cm) were cut from the margin of seven day-old colonies of a single conidium isolate of FG407. For each PDA plate, one plug was placed in the center with the mycelial side facing the agar. Plates were incubated in the dark at 24 ± 1 °C and 70% RH for six days. Subsequently, radial growth was determined by measuring the diameter of the fungal colony at two positions (smallest and largest diameter) and calculating the average of both values. Data are presented as percentage growth of the aqueous controls. The experiment was conducted two times and the results were pooled.

### 3.5. Climate Chamber Experiment—Reduction of Disease Severity and Mycotoxins in Artificially Inoculated Wheat

For these experiments, poly conidia isolates of FG0407, FG0410, FG9915 and FCr9703 were used. Suspensions with powder of the ABs GC, RP and FA were applied to wheat plants without any filtration. The methods for cultivation of the spring wheat cultivar “Apogee” (*Triticum aestivum* L.), inoculation of fungi, disease assessment and harvest were conducted according to Vogelgsang *et al.* [42]. One modification to this protocol was that for inoculation, the pots were transferred for 24 h in a walk-in climate chamber at 19–20 °C with 90% RH, followed by 48 h with 85% RH and a dark and light period of 9 h and 15 h, respectively. In addition, the TKW of harvested grains was assessed using a “Contador” seed counter (Baumann Saatzuchtbedarf, Waldenburg, Germany). The experimental set-up was a randomised complete block design. Each treatment consisted of eight pots (handled as four replicates with each two pots) with three wheat plants in each pot. “Apogee” is a full dwarf hard red spring wheat developed for life support system in space and is highly susceptible towards FHB [38,43].

The botanical powders were suspended in water with 0.1‰ Greemax^®^ (surfactant and adhesive emulsifier; Madora GmbH, Lörrach, Germany) [44] and stirred at room temperature for 2–3 h. All preparations and the water controls were applied with 37.5 mL per pot. TA and botanicals were applied with 5% suspensions, PrP with 0.375%. Two water controls with Greemax^®^ (0.1‰) amended tap water, whereof one was acidulated with acetic acid to pH 4.0, were used (see Table 1). For application of the botanicals, the pots were placed on a turntable and the heads of the wheat plants were sprayed from all sides until run-off. After application, the wheat heads were allowed to dry during 2 h. Visual disease assessments were conducted three times within 7–14 days post-inoculation by counting spikelets from all heads in each pot with symptoms and estimating the percentage of the diseased area. The experiment was conducted twice and the results were pooled.

For determination and quantification of mycotoxins, liquid chromatography tandem mass spectrometry (LC-MS/MS) analysis was used. 10 g wheat flour was placed in a 100-mL flask and 40 mL of an acetonitrile/acetone/water mixture 50:25:25 (v/v) (all organic solvents from Scharlau Multisolvent, Sentmenat, Spain; water from Gradient A10, Millipore, Bedford, MA, USA) were added. Closed flasks were manually agitated until no larger wheat flour aggregates were visible. The extraction was conducted on a rotary shaker (Bühler SM-30, Hechingen, Germany) for 2 h (180 rev min^−1^). Flour and solvent mixture were separated over a folded filter (Whatman 595½, Dassel, Germany), and the extract was collected in a 22-mL vial with a solid screw cap (Supelco, Bellefonte, PA, USA). Matrix components including lipids or fat were removed by cleaning 1 mL of extract over a 3 mL cartridge (Isolute, Uppsala, Sweden) filled with 0.15 g of celite (Fluka, 545 coarse, Buchs, Switzerland)/alox (Fluka, for chromatography, Buchs, Switzerland) 1:1 (w/w), wetted and precleaned with 2 mL of the same solvent mixture used for extraction. The resulting extract was collected in a 5-mL Reacti-vial (Supelco). After percolation of 1-mL extract, the cartridge was rinsed with 2 mL of solvent mixture and emptied by use of vacuum. The final volume of the cleaned extract (3 mL) was reduced at 40 °C to 0.4 mL with compressed air and transferred into a 2 mL high-performance liquid chromatography (HPLC)-vial. The Reacti-vial was rinsed with 0.4 mL water/methanol 90:10 during 10 s by the aid of a vortex (Scientific Industries, Bohemia, NY, USA) and transferred to the HPLC-vial as well. The final volume of the extract was adjusted with water/methanol 90:10 to 1 mL. The samples were stored in the dark at room temperature and were processed within 48 h. The LC-MS/MS analysis was performed on a Varian 1200-L system (Varian Inc., Walnut Creek, CA, USA). The analytes DON, NIV, acetylated-deoxynivalenol (Ac-DON: sum of 3-Ac- DON and 15-Ac-DON (all from R-Biopharm, Darmstadt, Germany) and ZEA (Sigma-Aldrich, St. Louis, MO, USA) were separated on a Polaris C18-A column (50 mm × 2.0 mm, 3 μm; Varian Inc., Walnut Creek, CA, USA). Operating the mass spectrometer in negative atmospheric pressure chemical ionization (APCI) mode, the analytes NIV, DON, Ac-DON and ZEA were detected with the next elution gradient: 0 min: 5% B (95% A); 1 min: 5% B; 4 min: 30% B; 5 min: 100% B; 12.5 min: 100% B; 13 min: 5% B; 20 min: 5% B. Eluent A consisted of water/methanol 95:5 (v/v) and eluent B of water/methanol 5:95 (v/v); both were buffered with 5 mL 1 M ammonium acetate per Liter (Fluka, Puriss P.A., Buchs, Switzerland). Each analyte was detected with two transitions (qualifier and quantifier) in multiple reactions monitoring (MRM). Analyte identification was confirmed using chromatographic retention time, correct mass of the mother ion, correct mass of the two daughter ions and agreement of the ratio of qualifier to quantifier with the calibration (±10%). For quantification, the method of matrix matching calibration was implemented to correct for eventual ion suppression. Recoveries for low (0.5 mg kg^−1^) and high (2 mg kg^−1^) spiked blank samples (*n* = 4) were between 86%–126% and 78%–107%, respectively. Method precision was in the range of 2%–12%, whereas instrument precision was between 2% and 10%. The limit of quantification for DON ranged between 0.09 mg kg^−1^ and 0.126 mg kg^−1^, for ZEA between 0.008 mg kg^−1^ and 0.021 mg kg^−1^ and for NIV between 0.058 mg kg^−1^ and 0.185 mg kg^−1^, depending on the sample series analyzed.

### 3.6. Field Experiments with Artificial Inoculation—Reduction of FHB and Mycotoxins in Wheat

Field experiments were conducted from the harvest years 2006–2010 with the two Swiss winter wheat cultivars “Runal” and “Levis”. According to the Swiss national catalogue, the resistance for FHB of these bread wheat cultivars is considered to be “medium” and “medium to poor”, respectively. The field experiments were carried out on the experimental farm of the Research Station Agroscope in Zurich-Reckenholz, Switzerland. The experiments comprised seven treatments (Table 1) with four replications each, using a Latin square design. The size of individual plots was 6.5 m × 2.6 m. Each plot was divided in two subplots for the two varieties sown in bands. Husbandry management was standard for the farm, except that no fungicides were applied.

For inoculation of the wheat plants, a suspension with a mixture of the four poly conidia isolates, FG0407, FG0410, FG9915 and FCr9703, was used. The suspension contained 2 × 10^5^ conidia mL^−1^ with equal amounts of each isolate and 0.125‰ Tween 20. The conidia mixture and a water control with 0.125‰ Tween 20 were applied from both sides along the field with a volume of 750 L ha^−1^ using a knapsack sprayer (width 1.5 m, 3 bar, Birchmeier M125, Birchmeier Sprühtechnik AG, Stetten, Switzerland). The inoculation was conducted at mid-anthesis (GS 63–65). The date for this growth stage varied considerably throughout the years: For example, in 2006, this stage was reached on 12 June, whereas in 2007 it was reached on 21 May. Wheat was inoculated from both sides along the plots, directing the spray towards wheat heads. Water and suspensions with ABs (GC, RP, FA) and TA were applied one day before the fungal inoculation and depending on weather conditions, again one or two days after the inoculation. For the application of TA, PrP and water, spray nozzles from TeeJet^®^ XR11002 (4.0 bar, 450 L ha^−1^) and for GC, RP and FA, spray nozzles from Floodjet^®^ (1.5 bar, 450 L ha^−1^) were used. Weather data were obtained from the MeteoSwiss operated weather station located at Zurich-Reckenholz.

Visual disease assessment from 4 × 10 randomly selected wheat heads within an individual plot was conducted in the field during two occasions between 14 and 25 days post-inoculation by counting spikelets with typical FHB symptoms and estimating the percentage of the diseased head area. Plots were combine-harvested when the cultivars reached GS 92 (caryopsis hard). Processing of the harvested samples, the procedure of a seed health test to determine the percentage incidence of FHB causing species and the preparation of samples for the analysis of toxins was done according to Vogelgsang *et al.* [42]. The method for the analysis of toxins was conducted as described above.

In 2007, there were no treatments with GC and RP in our field experiment with artificial infections and due to technical problems, the inoculation of wheat during anthesis was not possible. Hence, due to the resulting lack of orthogonally to the other years, the data of this experiment were not used for the analysis.

To determine the correlation of the field data with artificial inoculations, the results of 2007 were used but not those of 2008, because in 2008, the overall incidence by *Fusarium* species in wheat grains was close to 100% and hence, no seed health test was conducted.

### 3.7. Field Experiments with Semi-Natural Inoculation—Reduction of FHB and Mycotoxins in Wheat

To evaluate the performance of the ABs under conditions that mimic cropping of wheat without tillage after maize, *Fusarium* infected maize stubbles were applied in experimental field plots in 2010 after wheat emergence. The stubbles were inoculated in mid-November 2009 with FG/FCr suspensions of 1 × 10^6^ conidia mL^−1^, and stored in plastic boxes permeable to air in a greenhouse with an average temperature of 10 °C. The incubated stubbles were distributed in the field plots at the end of November 2009 (2–3 maize stalk pieces at about 0.5 kg m^−2^). Based on the indication of FG infection periods by FusaProg [9], the treatments were applied on 7 June or 7 and 8 June 2010, respectively (Figure 6). With the exception of the inoculation, all procedures and assessments of parameters were conducted as in the field experiment with artificial inoculations.

### 3.8. Statistical Analysis

For all experiments except for the one with semi-natural inoculations, results from the experimental runs were pooled in case of equal variances. In case of a failed normality or variance tests, data were arcsine, log or square root transformed before analysis of variance (ANOVA). Apart from one-way ANOVAs analysing the effect of one treatment factor only, two-way and three-way ANOVAs were also conducted for experiments where other factors than the botanicals were important (e.g., wheat cultivar and year). When the overall effect of the tested factor was significant in ANOVA, an all-pairwise multiple comparison procedure according to Holm-Sidak (α = 0.05) was employed. In order to specify the differences between treatments or years, a Tukey post hoc test (α = 0.05) was used. The variances of data from field experiments with artificial inoculations were not equal due to substantial year effects. Nevertheless, we conducted a three way ANOVA and a Tukey test since our experiments were orthogonal and the *p* value for the factor treatment was <0.0001 for all four criteria. In addition, separate mean values for the cultivars “Runal” and “Levis” and the corresponding standard error of means were calculated. For these ANOVAs, the open source “R” software version 3.0.1 (16 May 2013) was utilized. For calculation of Spearman correlations coefficients and for plotting of graphs from untransformed data, SigmaPlot version 11.0 (Systat Software) was used.

## 4. Conclusions

The overall aim of this study was to investigate possibilities to control FHB in wheat with substances having no negative effects on humans, animals and the environment. The obtained results prove that TA and the botanicals, GC and FA can substantially reduce FHB severity and mycotoxin contents under field conditions. In several experiments, the efficacy was even close to that observed with a synthetic fungicide.

In contrast to TA and GC, FA showed almost no fungal toxic effects *in vitro*. However, FA, being not effective *in vitro*, demonstrated great field performance in reducing the DON content in kernels by up to 71% under semi-natural inoculation conditions. The effectiveness of FA can best be explained by resistance inducing effects.

With a better understanding of the interactions between FA and wheat, such as the type and the dynamic of potentially induced compounds, the efficacy could be improved further. In addition, an optimized application strategy could be developed. The elicitor FA might be applied at the end of ear emergence. Subsequently, by using a forecasting system, which predicts FHB infection by FG and DON contamination, a second treatment with an antifungal product including TA or GC could be applied during flowering. With such an approach, it might be possible to obtain FHB and DON reductions as good as those from the best commercial fungicides.

Certainly, FHB in small-grain cereals can only be controlled with an integrated approach, employing crop rotation, tillage and proper choice of cultivars. However, data from our field experiments suggest that TA, GC and FA do have a high potential and thus could provide an excellent contribution to the production of safe small-grain cereals with acceptable toxin contents in low-input farming systems.
